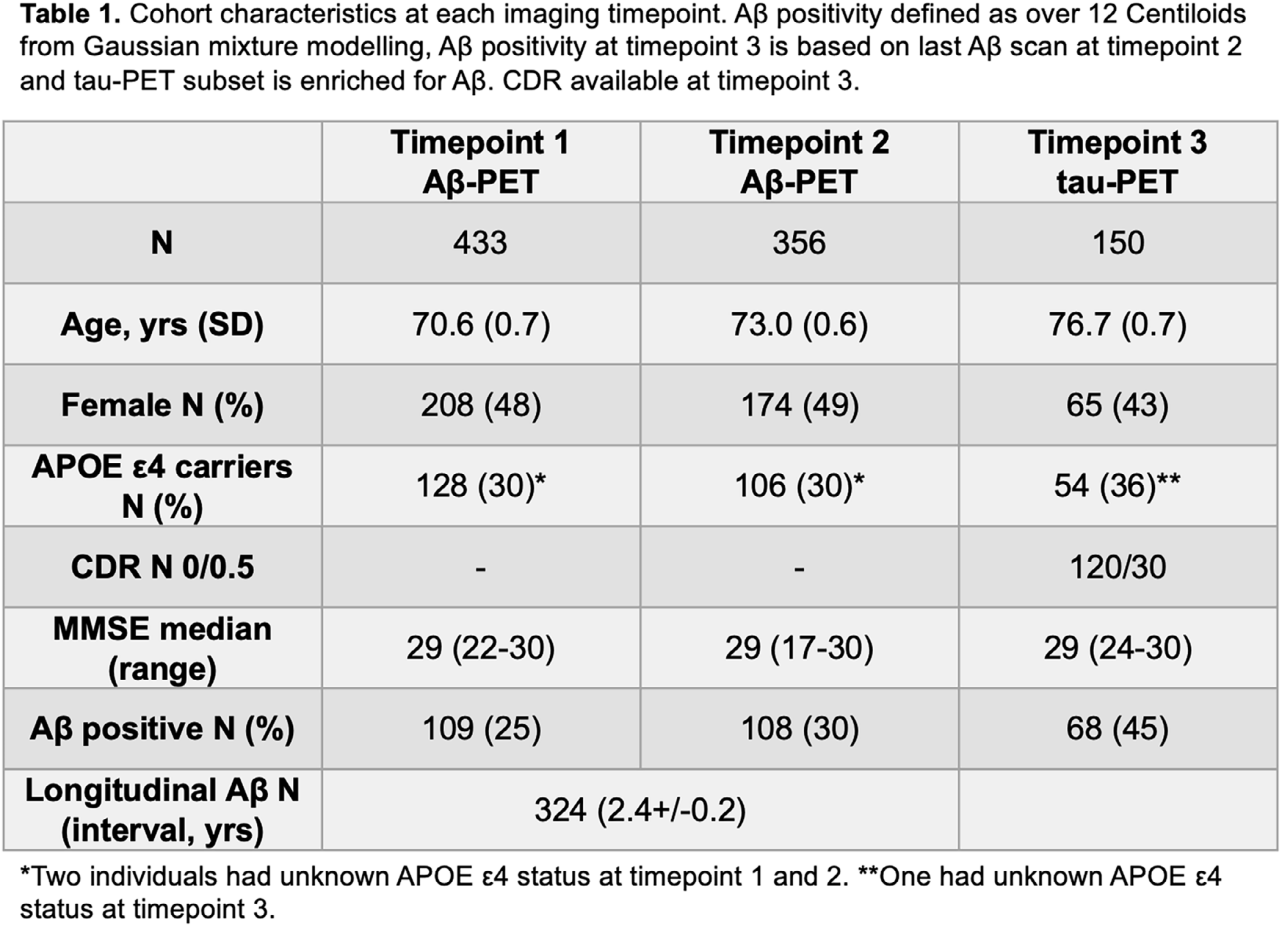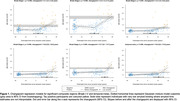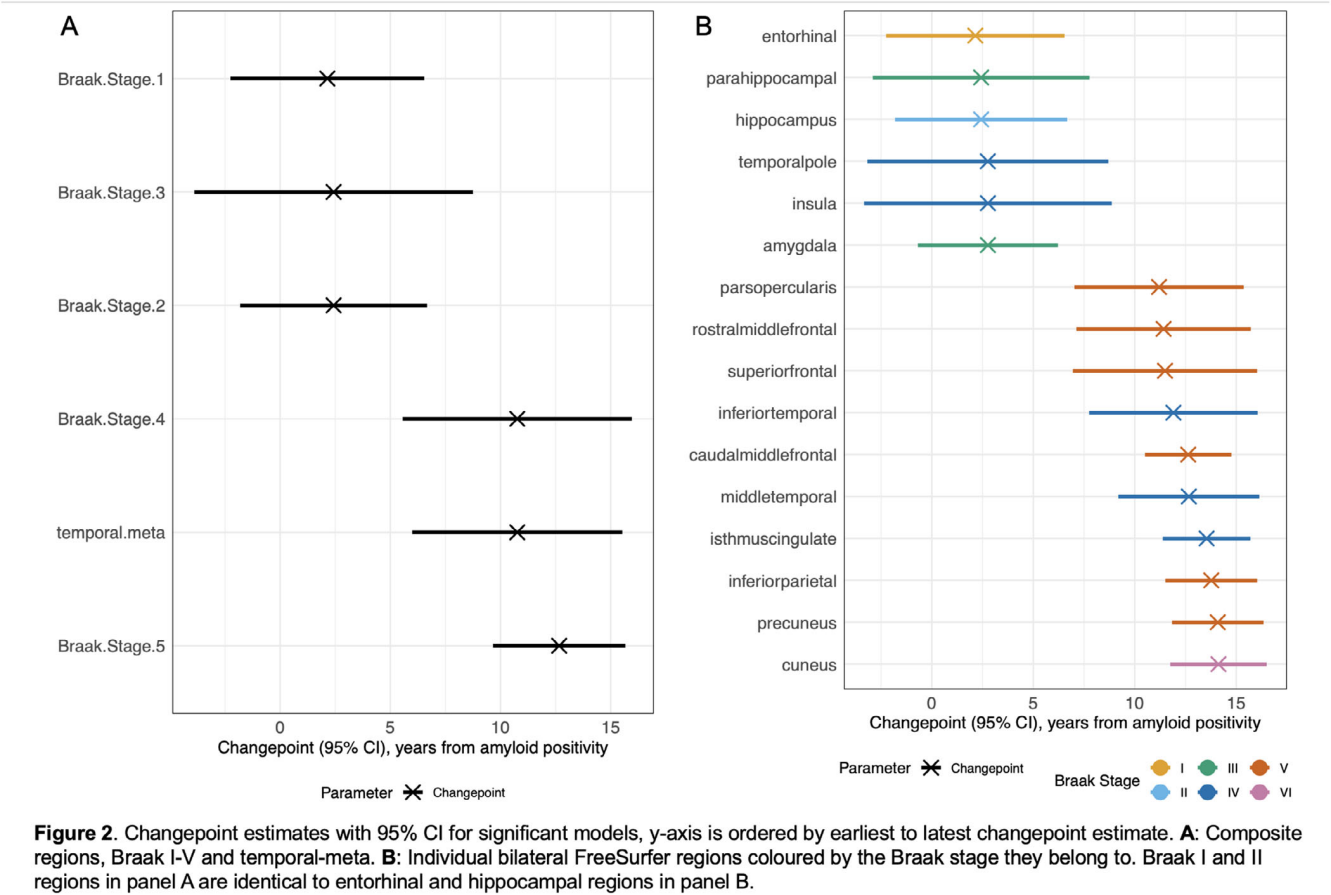# Estimating the time between Aβ positivity and elevated regional tau in preclinical AD

**DOI:** 10.1002/alz.092286

**Published:** 2025-01-09

**Authors:** William Coath, Tobey J. Betthauser, Jordan P Teague, Erik Arstad, Ramla Awais, Kerstin Sander, Catherine J Scott, David L Thomas, John Dickson, Pawel J Markiewicz, Frederik Barkhof, Michael Schöll, Marcus Richards, Nick C Fox, David M Cash, Jonathan M Schott, the Insight 46 team

**Affiliations:** ^1^ Dementia Research Centre, UCL Queen Square Institute of Neurology, University College London, London United Kingdom; ^2^ Alzheimer’s Disease Research Center, University of Wisconsin‐Madison, Madison, WI USA; ^3^ University of Wisconsin‐Madison School of Medicine and Public Health, Madison, WI USA; ^4^ Department of Medicine, University of Wisconsin‐Madison School of Medicine and Public Health, Madison, WI USA; ^5^ Wisconsin Alzheimer’s Disease Research Center, University of Wisconsin‐Madison, Madison, WI USA; ^6^ Radiopharmaceutical Chemistry, University College London, London United Kingdom; ^7^ Institute of Nuclear Medicine, University College London Hospitals, London United Kingdom; ^8^ Dementia Research Centre, UCL Queen Square Institute of Neurology, London United Kingdom; ^9^ Neuroradiological Academic Unit, Department of Brain Repair and Rehabilitation, UCL Queen Square Institute of Neurology, University College London, London United Kingdom; ^10^ Dementia Research Centre, Department of Neurodegenerative Disease, UCL Queen Square Institute of Neurology, University College London, London United Kingdom; ^11^ Wellcome Centre for Human Neuroimaging, UCL Queen Square Institute of Neurology, London United Kingdom; ^12^ UCL Institute of Nuclear Medicine, London United Kingdom; ^13^ UCL Centre for Medical Image Computing, London United Kingdom; ^14^ Institutes of Neurology and Healthcare Engineering, University College London, London United Kingdom; ^15^ Amsterdam Neuroscience, Vrije Universiteit Amsterdam, Amsterdam UMC, Amsterdam Netherlands; ^16^ VU University Medical Center, Amsterdam Netherlands; ^17^ Department of Psychiatry and Neurochemistry, Institute of Neuroscience and Physiology, The Sahlgrenska Academy, University of Gothenburg, Mölndal Sweden; ^18^ MRC Unit for Lifelong Health & Ageing at UCL, London United Kingdom; ^19^ UK Dementia Research Institute at UCL, London United Kingdom

## Abstract

**Background:**

Understanding when Aβ positive cognitively normal individuals develop tau pathology has important implications for treatment with anti‐Aβ therapies. We employed a changepoint regression approach to estimate time from Aβ‐PET positivity to regionally elevated tau‐PET in a population‐based cohort of primarily cognitively unimpaired individuals.

**Method:**

Participants from Insight 46 (1946 British birth cohort) underwent two [^18^F]florbetapir Aβ‐PET scans and a sub‐sample enriched for Aβ were also scanned with [^18^F]MK‐6240 tau‐PET, characteristics are presented in Table 1. Age at Aβ‐PET positivity, defined through Gaussian mixture modelling (GMM) as >12 Centiloids, was estimated using the SILA algorithm (Betthauser et al. 2022). Tau‐PET SUVRs from a temporal‐meta, Braak I‐VI and 34 bilateral FreeSurfer7 regions were calculated 90‐110 mins post‐injection using an inferior cerebellar grey matter reference region. Tau‐PET positivity cutpoints for each region were defined using the 99^th^ percentile of the lower distribution from GMM. For each region, we tested changepoints in the slope of the linear regression between tau‐PET SUVR and time from estimated Aβ positivity with Bonferroni correction (5% significance, p<.001).

**Result:**

At tau‐PET scan, 90 individuals (60%) were >0 years from estimated Aβ positivity. There was a statistically significant difference in slopes before and after the changepoint for all composite regions excluding Braak VI (Figure 1). Changepoint estimates show that tau accumulation started ∼2.5yrs after estimated Aβ positivity in Braak stages I‐III; and ∼10‐12.5yrs for Braak stages IV‐V, and the temporal‐meta region (Figure 2A). Regional estimates broadly followed Braak stages for 16/36 regions showing significant evidence of change in slopes (Figure 2B). Tau positivity rates before changepoints did not exceed 5%, whereas post‐changepoint tau positivity rates were between ∼10% and ∼50%. Regions with later changepoints tended to exhibit higher post‐changepoint slopes with greater uncertainty.

**Conclusion:**

Elevated tau‐PET signal occurs ∼2.5 years after estimated age of Aβ onset in Braak stages I‐III and ∼10‐12.5 years in temporal‐meta and Braak stages IV‐V, with uncertainty within these groupings. These estimates for later Braak stages are consistent with results from previous work on the timing of positive tau PET visual reads. These findings may have implications for determining the optimal time to administer Aβ immunotherapies in asymptomatic individuals.